# The effect of downregulation of Stathmin gene on biological behaviors of U373 and U87-MG glioblastoma cells

**DOI:** 10.1186/s40659-018-0160-0

**Published:** 2018-06-08

**Authors:** Ping Liu, Junyan Yu, Xiangyang Tian, Jianlan Chang, Ying Zhang, Rong Zhang, Ningning Zhang, Ranxing Huang, Lulu Li, Xianli Qiao, Hongliang Guo

**Affiliations:** Department of Oncology, Peace Hospital of Changzhi Medical College, Changzhi, 046000 Shanxi China

**Keywords:** Stathmin, Glioblastoma, Cell proliferation, Cell migration, Cell cycle, Tumorigenicity

## Abstract

**Background:**

Stathmin as a critical protein involved in microtubule polymerization, is necessary for survival of cancer cells. However, extremely little is known about Stathmin in glioblastoma. So, this study was designed to elucidate the function of Stathmin gene in the tumorigenesis and progression of glioblastoma cells.

**Method:**

The lentiviral interference vector pLV3-si-Stathmin targeting Stathmin gene and the control vector pLV3-NC were established for the co-transfection of 293T cells together with the helper plasmids. Viral titer was determined via limiting dilution assay. Then pLV3-si-Stathmin and pLV3-NC were stably co-transfected into U373 and U87-MG glioblastoma cells. Expression levels of Stathmin protein in each group were determined by using Western Blot, and the proliferation and migration ability of the cells with downregulated Stathmin were evaluated through CCK8 assay and transwell invasion assay, respectively. Cell cycles and cell apoptosis were detected with flow cytometry. Finally, the effect of Stathmin in tumor formation was determined in nude mice.

**Result:**

DNA sequencing and viral titer assay indicated that the lentiviral interference vector was successfully established with a viral titer of 4 × 10^8^ TU/ml. According to the results from Western Blotting, Stathmin protein expression level decreased significantly in the U373 and U87-MG cells after transfected with pLV3-si-Stathmin, respectively, compared with those transfected with pLV3-NC. In glioblastoma cells, the cell proliferation and migration were greatly inhibited after the downregulation of Stathmin protein. Flow cytometry showed that much more cells were arrested in G2/M phasein Stathmin downregulated group, compared with the non-transfection group and NC group. But Stathmin downregulation did not induce significant cell apoptosis. Tumor formation assay in nude mice showed that tumor formation was delayed after Stathmin downregulation, with a reduction in both tumor formation rate and tumor growth velocity.

**Conclusion:**

Stathmin downregulation affected the biological behaviors of U373 and U87-MG glioblastoma cells, inhibiting the proliferation and migration of tumor cells. Stathmin gene may serve as a potential target in gene therapy for glioblastoma.

## Background

Glioblastoma is the most common intracranial malignant tumor and originates from the neural epithelia. Approximately 80% of patients with neuroglioma are complicated with epilepsy [1]. So far, the therapeutic effect on glioblastoma, especially high-grade glioblastoma, remains unsatisfactory.

Microtubule regulatory protein Stathmin, with a molecular weight of 17 kDa, is highly conserved and plays an important role in regulating the dynamic equilibrium of microtubule system [2]. Stathmin is involved in the assembly and regulation of microtubules and spindles through binding to the tubulin. Besides, it also participates in cell proliferation, differentiation, regeneration and motion via binding to different proteins [3]. Stathmin is a key regulator of the signaling pathway, thus affecting the cell growth. Up-regulated Stathmin has been detected in various human tumors, including breast cancer, lung cancer, cervical cancer, esophageal cancer, endometrial cancer and liver cancer [4–8]. Therefore, it is plausible to make Stathmin as a target in gene therapy for tumors. Liu and Akhtar et al. had already shown that Stathmin downregulation inhibited the cell migration and cell proliferation in gastric cancer cells [9, 10], and Marie observed that Stathmin was increased in glioblastoma and a strong correlation between MELK and Stathmin expressions in glioblastoma clinical samples [11]. In the present study, we demonstrated that downregulation of Stathmin expression inhibited the cell proliferation, cell migration and tumor formation in nude mice of U373 and U87-MG glioblastoma cells. Furthermore, knockdown of the Stathmin expression also induced the cell cycle arrest at G2/M phase, but no significantly statistical difference on apoptosis rate in U373 and U87-MG cells. So, Stathmin affects glioblastoma progression through regulating cell proliferation, cell cycle and cell migration. Our finding demonstrates that Stathmin gene might serve as a potential therapeutic target in glioblastoma.

## Results

### Downregulation of the Stathmin expression in U373 and U87-MG glioblastoma cells

**Fig. 1:**
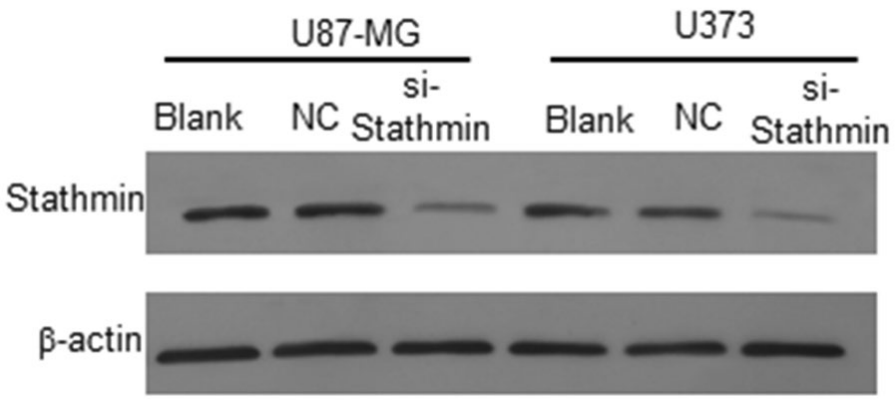
The expression of Stathmin protein in U373 and U87-MG cells by various treatment. Western blot result showed that Stathmin protein was significantly decreased in pLV3-si-Stathmin group compared with untransfected blank group and pLV3-NC group

To study the role of Stathmin in glioblastoma, the expression of the Stathmin was knockdown by siRNA-Stathmin in U373 and U87-MG glioblastoma cells. Firstly, we constructed the pLV3-NC and pLV3-si-Stathmin vectors, and obtained the pLV3-NC and pLV3-si-Stathmin lentivirus to transfect the glioblastoma cells. The U373 and U87-MG cells were transfected with the pLV3-NC and pLV3-si-Stathmin lentivirus, respectively, after 72 h, the blank cells and the transfected cells were collected for Western blot. As shown in Fig. 1, Stathmin was downregulated in U373 and U87-MG cells after transfected with pLV3-si-Stathmin for 72 h. Based on these results, the expression of Stathmin protein was downregulated in U373 and U87-MG cells by pLV3-si-Stathmin lentivirus.

### Downregulation of Stathmin expression affects the proliferation of U373 and U87-MG cells

**Fig. 2:**
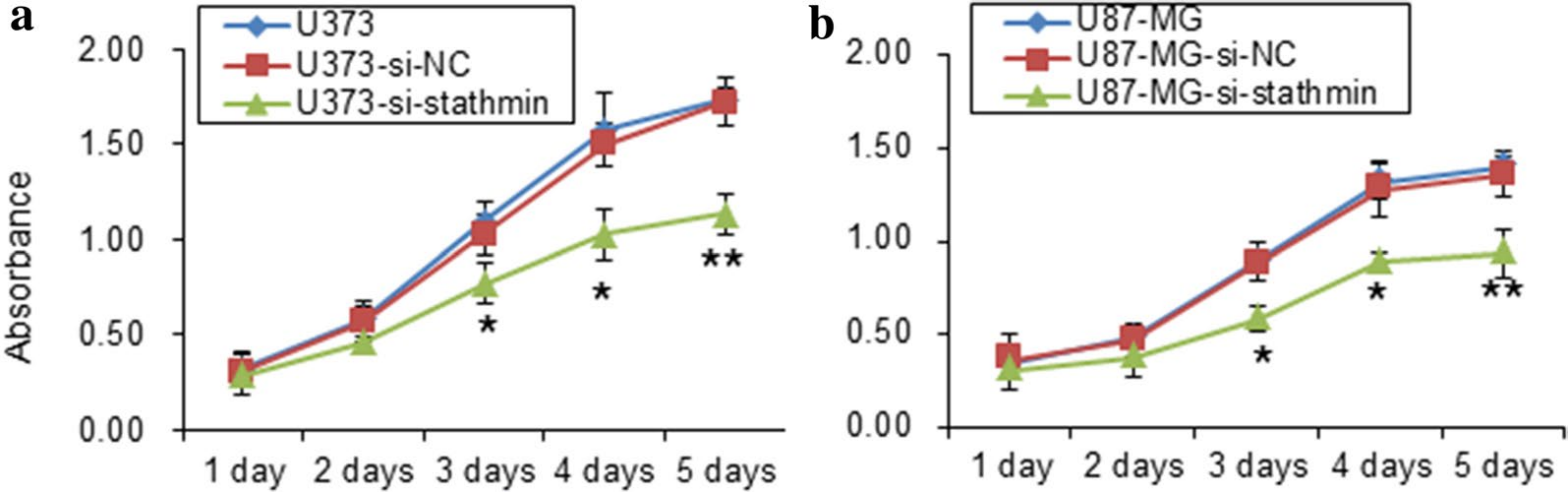
Proliferation assay of U373 and U87-MG cells through various treatments. Growth curves of U373 cell (**a**) and U87-MG cell (**b**) from 1 to 5 days with three treatments (untransfected control, pLV3-NC transfected group and pLV3-si-Stathmin transfected group) detected through CCK-8 assay

To study the role of *Stathmin* in cell proliferation ability, we performed the transfection of U373 and U87-MG cells by pLV3-si-Stathmin. Cell viability was measured with CCK8 assay after transfection for the indicated time. As shown in Fig. 2a, b, all of the blank cells and the cells transfected with pLV3-NC and pLV3-si-Stathmin lentivirus were growing during 1–5 days. However, the cells transfected with pLV3-si-Stathmin lentivirus significantly decreased (P < 0.05, one way ANOVA) when compared with the pLV3-NC and blank cells from 3rd to 5th day by CCK-8 detection (Fig. 2a, b). These results indicate that downregulation of Stathmin expression decreased the cell proliferation of U373 and U87-MG.

### Downregulation of Stathmin expression induces the cell cycle arrest of U373 and U87-MG cells

**Fig. 3:**
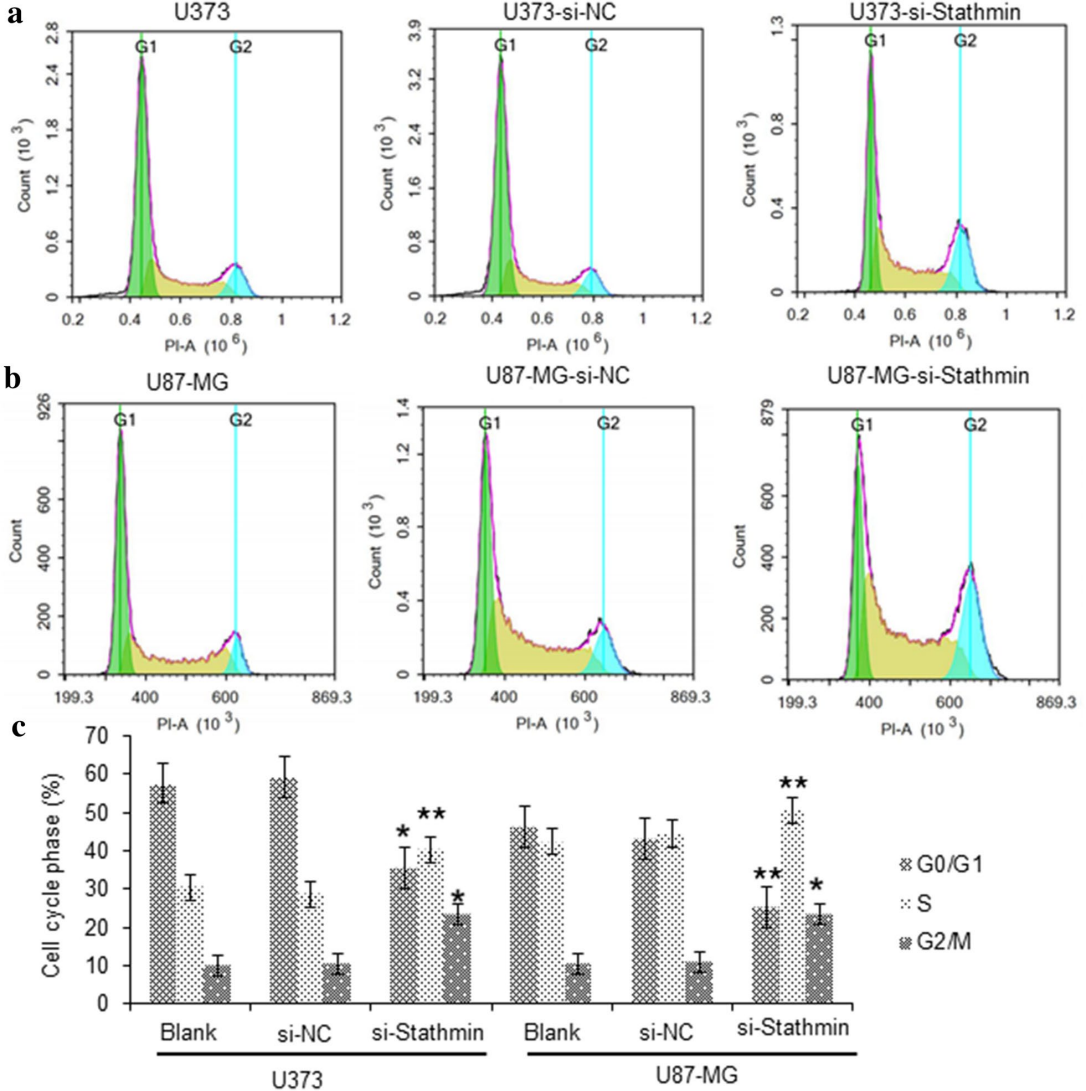
The distribution of cell cycle in U373 and U87-MG cells with different treatment. **a** The U373 cells with different treatment were analyzed applying flow cytometry. **b** The U87-MG cells with different treatment were analyzed applying flow cytometry. **c** Statistical analysis of Stathmin knockdown effect on cell cycle progression of U373 cells U87-MG cells *P<0.05, vs. negative control group; **P<0.01, vs. negative control group

To further elucidate the growth suppressing effect of Stathmin on U373 and U87-MG cells, we performed cell cycle distribution analysis using flow cytometry after the transfection of pLV3-si-Stathmin lentivirus for 72 h. The cell cycle analysis results demonstrated that downregulation of Stathmin induced G_2_/M phase arrest significantly in U373 and U87-MG cells (Fig. 3a, b). These results indicate that Stathmin expression is involved in the regulation of cell cycle in U373 and U87-MG cells.

### Knockdown of Stathmin was insignificant on apoptosis rate of U373 and U87-MG cells

**Fig. 4:**
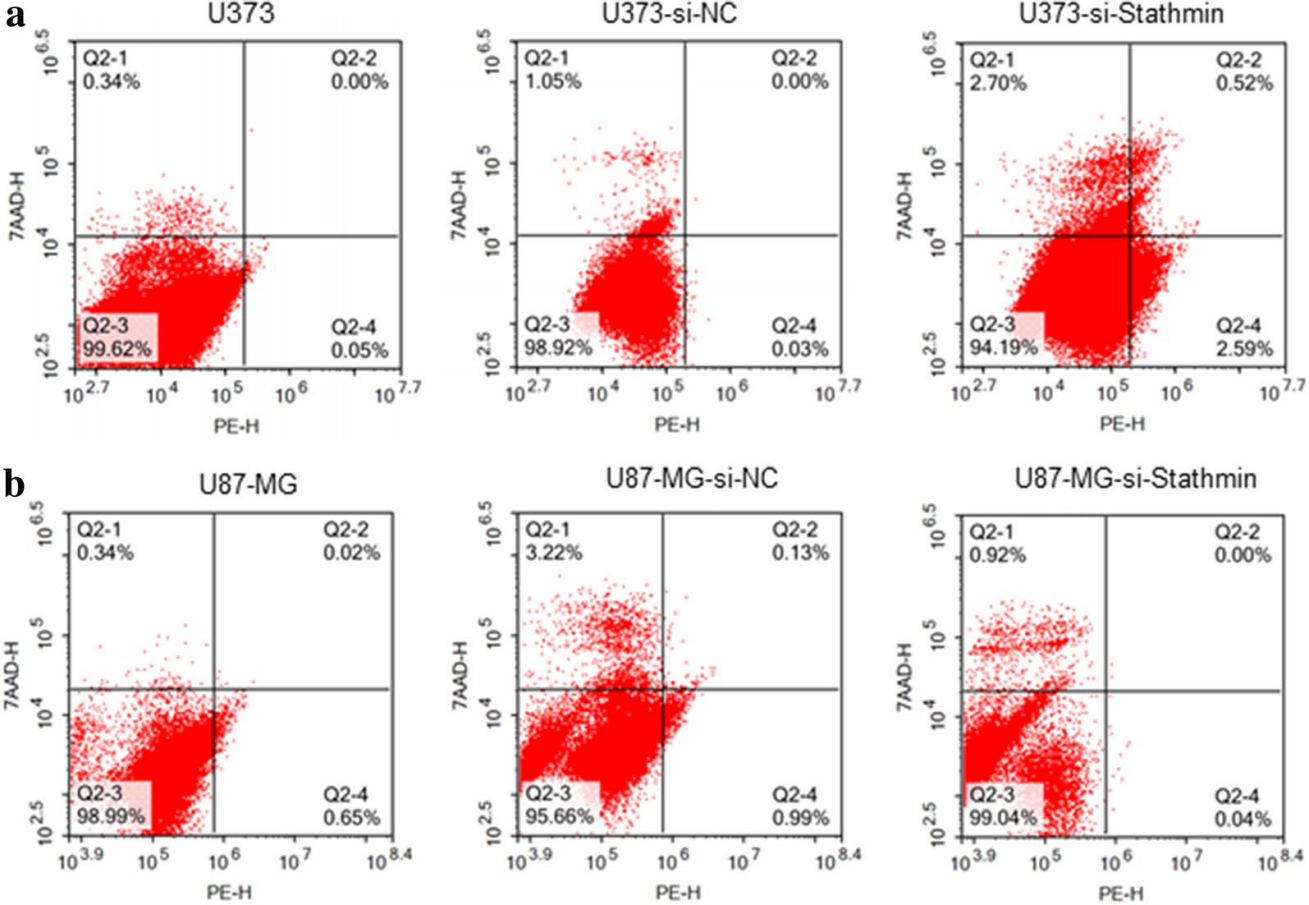
Assessment of Stathmin gene silencing on cell apoptosis. No difference in apoptosis rate was observed between pLV3-si-Stathmin group transfected group and pLV3-NC transfected group or untransfected blank group

To study the role of Stathmin on cell apoptosis, U373 and U87-MG cells were transfected by pLV3-si-Stathmin lentivirus for 72 h. Cell number of apoptosis was detected by flow cytometry. As shown in Fig. 4, the mean apoptosis rate of pLV3-si-Stathmin group, pLV3-NC group and blank group was not significant in U373 and U87-MG cells, respectively (P > 0.05).

### Downregulation of Stathmin expression inhibits the migration of U373 and U87-MG cells

**Fig. 5:**
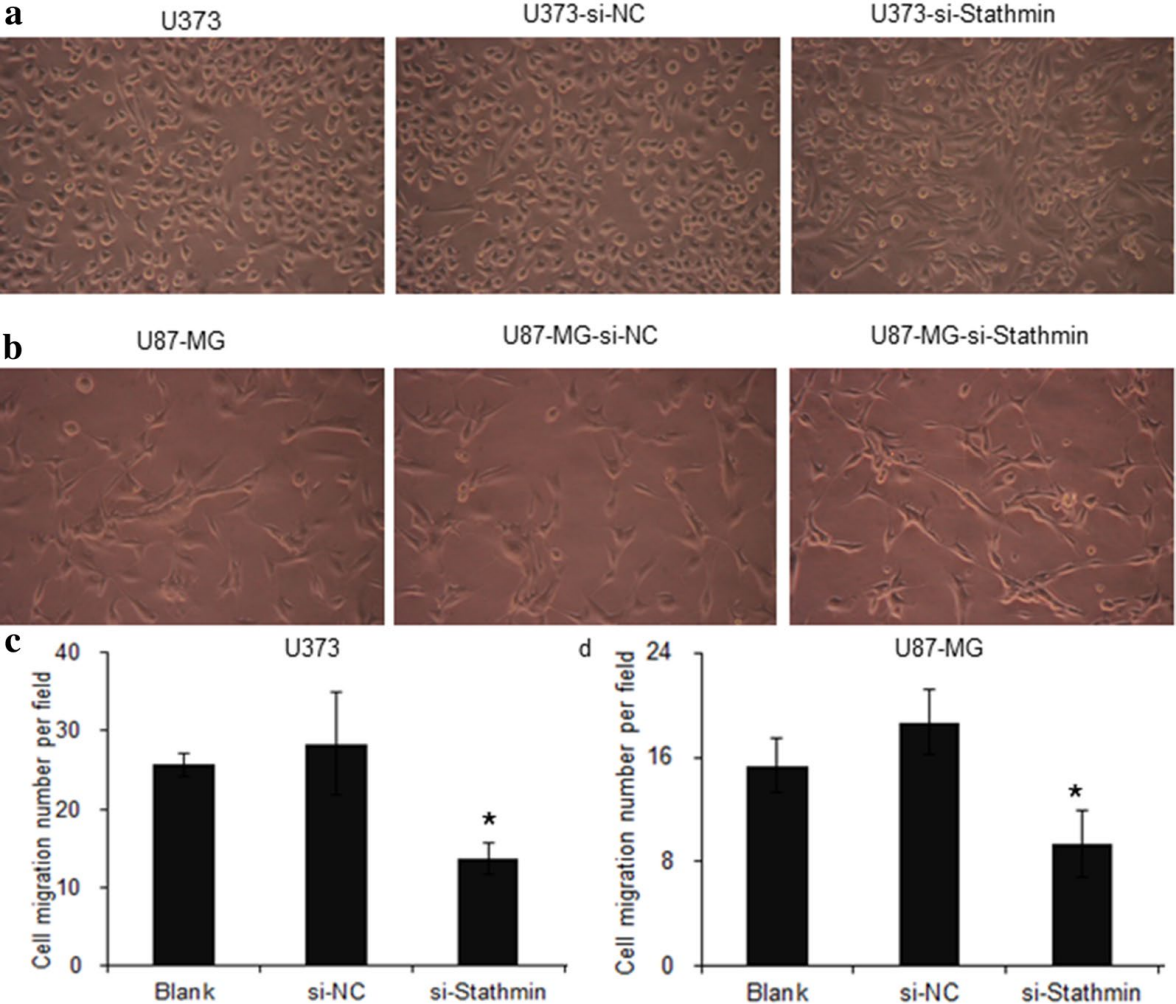
Transwell migration assay of U373 and U87-MG cells through various treatments. **a**, **b** Transwell migration assay was used to evaluate the migration ability of both U373 and U87-MG with three treatments. **c, d** The statistical analysis of Stathmin knockdown effect on cell migration of U373 cells U87-MG cells (untransfected control, pLV3-NC transfected group and pLV3-si-Stathmin transfected group). *P < 0.05, compared to untransfected control group

Stathmin plays an important role in modulation and microtubule polymerization, so it may affect the cell migration. To study whether Stathmin expression could affect the cell migration, we also carried the assay by downregulation of Stathmin. The U373 and U87-MG cells were transfected with pLV3-NC and pLV3-si-Stathmin lentivirus, and 72 h later, the cells were seeded to the transwell chamber, and the results showed in Fig. 5a, b. Transwell assays showed that Stathmin downregulation significantly inhibited the migration of U373 and U87-MG cells, and the inhibition rates were 53.09 ± 2.14% (P = 0.000) and 49.38 ± 7.71% (*P *= 0.00), respectively (Fig. 5c, d). These results indicated that knockdown of Stathmin expression could decrease the migration ability of U373 and U87-MG cells.

### Downregulation of Stathmin expression inhibits tumorigenicity in nude mice

**Fig. 6:**
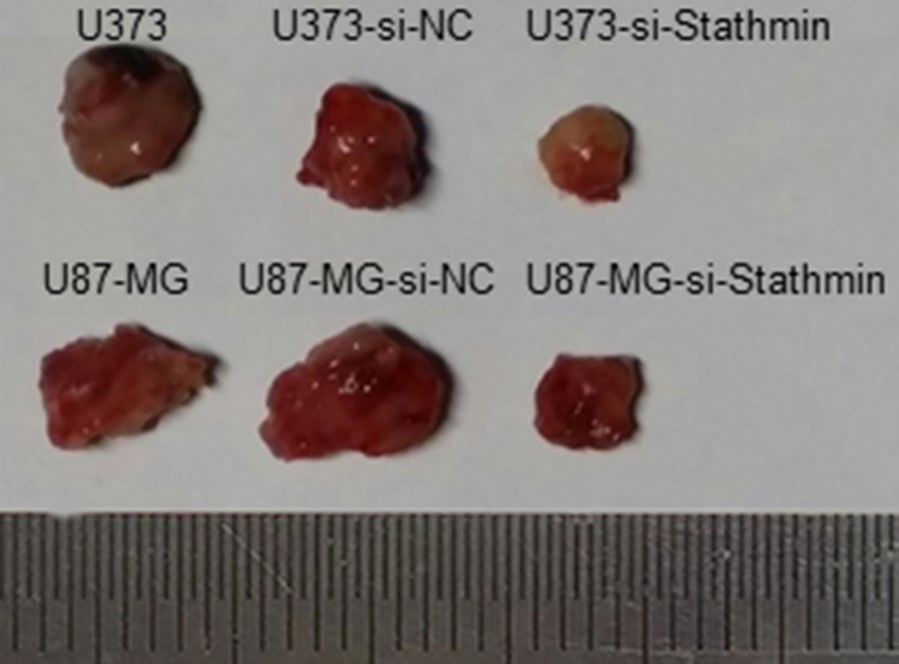
Assessment of Stathmin knockdown on tumorigenicity in nude mice. Photograph of xenografts dissected from nude mice 12 weeks after subcutaneous inoculation for different treatment, the transfection of pLV3-si-Stathmin suppressed the growth of U373 and U87-MG cells compared to pLV3-NC transfected group or untransfected blank group

To determine the contribution of Stathmin to tumor development of glioblastoma cells in vivo, we performed orthotopic injections of U373 and U87-MG cells in mammary fat pads of nude mice. The blank cells, pLV3-si-Stathmin or pLV3-NC lentivirus transfection cells were injected into the mice respectively, and all nude mice were sacrificed 12 weeks of initial implantation. As shown in Fig. 6, tumor growth was significantly slower in mice injected with the pLV3-si-Stathmin cells than those injected with the pLV3-NC cells or the blank cells of U373 and U87-MG cells (P < 0.01). The volume and weight of the tumors are shown in Table 1. These data show that downregulation of Stathmin expression inhibits tumor formation of U373 and U87-MG cells in nude mice.

**Table 1:** Tumor formation induced by transplanting U373 and U87-MG cells transfected with positive vector

Group	Tumor formation at 12 w after inoculation		
	Tumor formation rate	Volume (mm^3^)	Weight (g)
U373	6/6	879 ± 80	6.23 ± 1.45
U373-NC	6/6	825 ± 78	5.56 ± 1.20
U373-si-Stathmin	4/6	359 ± 56*^,#^	2.71 ± 1.02*^,#^
U87-MG	6/6	790 ± 89	5.37 ± 1.51
U87-NC	6/6	885 ± 95	6.69 ± 1.27
U87-si-Stathmin	4/6	321 ± 59*^,#^	2.63 ± 0.65*^,#^

* P < 0.01, compared to blank control group

^#^P < 0.01, compared to NC group

## Discussion

Human Stathmin gene, also known as oncoprotein 18 and mapped to chromosome 1P36.11, acts as an important microtubule regulatory protein [12]. Several studies have demonstrated that Stathmin expression is associated with the pathology and poor prognosis of several tumors including the brain, oral mucosa, breasts, ovaries, cervix and melanoma [13–18]. Stathmin overexpression increases the malignancy and migration of tumors, thus reducing the overall survival. Suppressing Stathmin can delay tumor progression through changing cell cycle and phenotype [19].

RNA interference (RNAi) is an emerging technique in gene therapy of tumors [20]. Since it is hard to transfect some cells, like primary cells, stem cells and undifferentiated cells, and lentiviral vector can effectively deliver the exogeneous gene or short hairpin RNA (shRNA) to host chromosome, the use of these vectors can increase the transfection of target genes or shRNAs. RNAi using lentiviral vectors are reported to be able to reduce the expression of lung cancer gene [21] and oncogenicity of liver cancer [22]. In this study, we also used lentiviral vectors to deliver Stathmin containing plasmid into U373 and U87-MG glioblastoma cells. Also, we confirmed the knockdown efficiency by Western Blot, and the results showed that Stathmin expression was inhibited significantly (Fig. 1).

It is generally agreed that Stathmin downregulation will interfere with the biological behaviors of tumors. Feng employed RNAi technique to induce Stathmin downregulation in esophageal cancer cells EC9706 and Eca-109, and found that the proliferation of the cells decreased with more cells arrested in G2/M phase or undergoing apoptosis [23]. Song induced Stathmin downregulation in glioblastoma cells U251 and U87-MG, and observed the inhibiting of cell proliferation, migration and invasion [24], also showing similar results as that in the study by Feng [23]. Additionally, Li, Akhtar and Zhang all found similar results in pancreatic cancer, gastric cancer and breast cancer, respectively [12, 25, 26].

Proliferation and migration are two basic features of tumor cells. Through transfecting si-Stathmin lentivirus into U373 and U87-MG cells, the current study decreased the proliferation and migration of these two cells (Figs. 2a, b and 5a, b). CCK8 assay and transwell invasion assay demonstrated that Stathmin downregulation significantly inhibited the proliferation and migration of U373 and U87-MG cells compared with the blank cells and the cells transfected with NC. These results indicated from another perspective that Stathmin gene could promote the proliferation and migration of U373 and U87-MG cells, as reported by previous literatures.

Loss of control of the cell cycle is one of the critical steps in the development of cancer. PI staining combined with flow cytometry was performed in our study to determine the effect of Stathmin on cell cycle distribution of U373 and U87-MG cells. The results displayed that U373 and U87-MG cells transfected with si-Stathmin lentivirus were arrested more in G2/M phase (Fig. 3a, b), while the number of cells arrested in G1 and S phase was decreased significantly. Thus Stathmin gene silencing successfully induced cell cycle arrest, which agreed with findings from most previous experiments. In other words, Stathmin gene played an important role in promoting cell cycle progression for U373 and U87-MG cells.

Additionally, annexin V-PE-7-AAD apoptosis kit was further used and the results showed that transfection of si-Stathmin lentivirus did not substantially affect the apoptosis of U373 and U87-MG cells (Fig. 4), which contradicted previously relevant findings probably due to the use of different cell lines.

Tumor formation experiment in nude mice was also performed to determine tumor cell proliferation in vivo. After silencing Stathmin, the tumor formation rate was decreased significantly and the cells grew less actively (Fig. 6 and Table 1). Therefore, downregulating Stathmin could inhibit tumor formation induced by inoculating U373 and U87-MG cells. The proliferation and migration of U373 and U87-MG cells were significantly reduced after Stathmin downregulation. Moreover, Stathmin downregulation could promote cell cycle arrest and reduction in oncogenicity.

In conclusion, we provide an early report that the role of Stathmin in the tumorigenesis and progression of glioblastoma. Our findings indicate that knockdown of Stathmin expression leads to inhibiting of cell proliferation, tumor formation and cell migration, also inducing cell cycle arrest in U373 and U87-MG glioblastoma cells. Consequently, Stathmin might serve as a novel diagnosis and prognosis biomarker as well as a potential therapeutic target in glioblastoma.

## Methods

### Reagents and antibodies

Primers, plasmid extraction kit and gel recovery kit were purchased from Shanghai Bio-engineering (Shanghai, China). Lipofectamine 2000 reagent was purchased from Invitrogen (Carlsbad, CA, USA). Fetal bovine serum (FBS) was purchased from Hyclone (Logan, USA). Electrochemiluminescence (ECL) reagent was purchased from Millipore (Billerica, MA, USA). RIPA lysis buffer, protease inhibitor and BCA protein assay kit were purchased from Beyotime Institute of Biotechnology (Jiangsu, China). Stathmin primary antibody was purchased from Abcam (Abcam, UK). Goat anti-rabbit/mouse secondary antibody was purchased from Jackson.

### Cell lines and plasmid

U373 and U87-MG glioblastoma cells and 293T cells were obtained from American Type Culture Collection (Manassas, VA, USA). Glioblastoma cells were cultured in DMEM-H medium containing 10% FBS, 100 U/ml penicillin and 100 μg/ml streptomycin, and placed in a 5% CO_2_ incubator at 37 °C. The cells were digested with 0.25% trypsin and passaged every 2–3 days. Log-phase cells were harvested for experiment. Lentiviral vector pGLV3/H1/GFP + Puro were purchased from GenePharma (Shanghai, China).

### Lentivirus packaging and infection

The generation of the pLV3-si-Stathmin and control lentivirus was conducted according to a protocol described in Akhtar’s paper. The siRNA sequence was 5′-TTATTAACCATTCAAGTCC-3′ reported by Akhtar [10], and NC sequence was 5′-TTCTCCGAACGTGTCACGT-3′. The loop sequence of the pGLV3 template was TTCAAGAGA to avoid the stop codon. This fragment was transferred into the chromosome of *E. coli* after sequencing. Plasmid extraction was performed on correct clones.

The lentiviral vector pLV3-si-Stathmin and the helper plasmid pGag/Pol, pRev and pVSV-G were co-transfected into the 293T cells using lipofectamine 2000 reagent. The transfected cells were cultured at 37 °C in a 5% CO_2_ incubator for 48–72 h, and then the green fluorescence was observed. The cells were harvested and the viral titer was determined via limiting dilution assay. U373 and U87-MG glioblastoma cells were infected with pLV3-NC and pLV3-si-Stathmin lentivirus. After gentle mixing, the cells were cultured at 37 °C in a 5% CO_2_ incubator for 48 h and then photographed.

### Western Blot analysis

The blank cells and the cells transfected with pLV3-NC and pLV3-si-Stathmin lentivirus were collected after culturing 72 h. Total protein extraction was performed using RIPA lysis buffer and the protein was quantified by the BCA Protein Assay Kit. Total protein 40 μg per lane was separated on a 12% SDS-PAGE gel and transferred to nitrocellulose membranes. Membranes were blocked by 5% skim milk in TBST for 2 h, and then incubated with the primary antibodies anti-Stathmin (1:1000) and anti-β-actin at 4 °C overnight. The membranes were washed and incubated with HRP-conjugated secondary antibody (1:4000) at room temperature for 2 h. The membranes were exposed in a dark room.

### CCK8 cell proliferation assay

Cell *proliferation* was determined by *CCK8* assay. The logarithmic phase cells were digested by trypsin to form a single cell suspension and seeded to the 96-well plates; and each sample had five plates and six replicates per plate. The plates were cultured at 37 °C in a 5% CO_2_ incubator for 1–5 days, respectively. Then, the cells were incubated in 10 μl of CCK8 solution for 2 h. The absorbance was measured for each well using a microplate reader at 450 nm at different time points.

### Transwell invasion assay

The blank cells and the cells transfected with pLV3-NC and pLV3-si-Stathmin lentivirus were collected after culturing 72 h. Then cells were coated to the 6-well transwell chamber with a density of 4 × 10^4^ and cultured at 37 °C in a 5% CO_2_ incubator for 48 h. The chamber was washed with 1 × PBS for three times. The cells were fixed in 4% paraformaldehyde for 20 min, washed with 1 × PBS for three times and transparentized with 0.1%Trition×-100 for 15 min. Then the cells were washed again with 1 × PBS for three times, stained with 100 μl of hematoxylin for 20 min and washed with running water for 2 min before soaked in fresh running water for 10 min. The membrane-penetrating cells were scrapped off and placed on the coverslip to dry. Finally, the coverslip was sealed with neutral balsam and observed under the microscope. For each sample 15 visual fields were selected randomly and the cells were counted within each visual field.

### Cell cycle analysis by flow cytometry Stathmin

The blank cells and the cells transfected with pLV3-NC and pLV3-si-Stathmin lentivirus were collected after culturing 72 h, and washed with PBS twice. The cells were fixed in 1 ml of 70% cold ethanol at 4 °C overnight. The cell precipitates were collected after removing fixing solution through centrifugation and cells were washed with PBS twice, and then incubated with 100 μl of 1 mg/ml RNaseA at 37 °C for 30 min. After the incubation, each tube was added into 300 μl PI dye and placed at room temperature in dark for 30 min. The cell cycle distribution was analyzed by flow cytometer and the fractions of cells in G0/G1, S, and G2/M phases were analyzed using Summit 5.0 software. The experiment was repeated three times.

### Stathmin cell apoptosis analysis by flow cytometry

The blank cells and the cells transfected with pLV3-NC and pLV3-si-Stathmin lentivirus were collected after culturing 72 h, then the culture medium was discarded, and the cells were washed with pre-cooled PBS twice and then collected after centrifuging at 1000 rpm for 5 min. The cells were suspended in 400 μl 1X binding buffer and incubated with 1 μl Annexin V-PE in dark for 15 min. After that, 5 μl of 7-AAD was added into each tube to further incubate the cells in dark at 2–8 °C for 5 min. Then the cells were detected by flow cytometer within 1 h after incubation and analyzed using Summit 5.0 software. Each sample was repeated three times.

### Tumor formation experiment in nude mice

The blank cells and the cells transfected with pLV3-NC and pLV3-si-Stathmin lentivirus were collected and inoculated into the nude mice at a dose of 1 × 10^7^; each treatment chose six nude mice. The tumor formation was recorded, and the length (L) and width (W) of each tumor were measured every week. All nude mice were sacrificed through cervical dislocation after 12 weeks of initial implantation. The transplanted tumors were harvested, and the tumor weight and volume were measured (tumor volume (V) = (length × width^2^) × 0.52).

### Statistical analysis

All statistical analyses were performed using SPSS 20.0 software. Data were reported as mean ± standard deviation (χ ± s). The difference was analyzed by one way ANOVA, P < 0.05 was considered statistically significant.

## Abbreviations

NC: negative control
CCK-8: Cell Counting Kit-8
RNAi: RNA interference
shRNA: short hairpin RNA
FBS: fetal bovine serum
ECL: electrochemiluminescence
PI: propidium iodide
SDS-PAGE: sodium dodecyl sulfate polyacrylamide gel electrophoresis
TBS: tris-buffered saline
